# Multicenter Study on the Impact of the Masker Babble Spectrum on the Acceptable Noise Level (ANL) Test

**DOI:** 10.3390/audiolres14060088

**Published:** 2024-12-07

**Authors:** Mark Laureyns, Giorgia Pugliese, Melinda Freyaldenhoven Bryan, Marieke Willekens, Anna Maria Gasbarre, Diego Zanetti, Julien Gilson, Paul Van Doren, Federica Di Berardino

**Affiliations:** 1 Audiology Department, Thomas More University College, 2800 Antwerp, Belgium; mark.laureyns@amplifon.com (M.L.);; 2 Amplifon Centre for Research & Studies, 20141 Milan, Italy; 3 Department of Clinical Sciences and Community Health, University of Milan, 20122 Milan, Italy; diego.zanetti@unimi.it (D.Z.); federica.diberardino@unimi.it (F.D.B.); 4 Department of Surgical Sciences, Audiology Unit, Fondazione IRCCS Ca’ Granda, Ospedale Maggiore Policlinico, 20122 Milan, Italy; annamaria.gasbarre@policlinico.mi.it; 5 School of Communication Science & Disorders, Louisiana Tech University, Ruston, LA 71272, USA; melinda@latech.edu; 6 Audiology Department, Marie Haps University College, 1050 Brussels, Belgium

**Keywords:** acceptable noise level, ANL test, noise tolerance in hearing impairment, hearing impairment ANL, ANL test masker babble spectrum

## Abstract

Introduction: Acceptable Noise Level (ANL) is defined as the most comfortable level (MCL) intensity for speech and is calculated by subtracting the maximum noise tolerable by an individual. The ANL test has been used over time to predict hearing aid use and the impact of digital noise reduction. This study analyzes this impact by using different masker babble spectra when performing the ANL test in both hearing-impaired and healthy subjects in three different languages (Dutch, French, and Italian). Materials and Methods: A total of 198 patients underwent the ANL test in their native language using a standardized protocol. The babble file was speech-weighted to match the long-term spectrum of the specific ANL language version. ANL was proposed in three different masking conditions: with multitalker Matched babble speech noise, with the same masking signal with the spectrum reduced from 2 kHz onwards (High Cut), and with the spectrum increased from 2 kHz onwards (High Boost). Results: In all of the comparisons among the three languages, ANL with High Boost noise gave significantly higher (worse) scores than ANL with Matched noise (*p*-value S1: <0.0001, S2: <0.0001, S3: 0.0003) and ANL with High Cut noise (*p*-value S1: 0.0002, S2: <0.0001, S3: <0.0001). The ANL values did not show any significant correlation with age and gender. In French, a weak correlation was found between ANL with High Cut noise and the Fletcher index of the worst ear. In Italian, a weak correlation was found between both ANL with Matched and High Boost noise and the Fletcher index of the best ear. Conclusions: ANL with High Boost added to noise stimuli was less acceptable for all patients in all of the languages. The ANL results did not vary in relation to the patients’ characteristics. This study confirms that the ANL test has potential application for clinical use regardless of the native language spoken.

## 1. Introduction

The first version of the Acceptable Noise Level (ANL) test was developed in 1991 by Nabelek et al. [1]. ANL is calculated by defining the most comfortable level (MCL) intensity for continuous speech selected by an individual and subtracting the maximum background noise level (BNL) that the individual is willing to put up with while listening to the speech. The first objective of the ANL test was to predict hearing aid use [2]. In 2013, Eddins et al. and, in 2019, Shetty et al. demonstrated that ANL is also a predictor of the impact of digital noise reduction (DNR) when using hearing aids [3,4]. Aided ANLs were significantly better (lower) with DNR for subjects with high (poor) ANLs but not for subjects with low (good) ANLs.

Most of the literature regarding ANL shows that ANL does not relate to the grade of hearing loss, age, and gender. In 2012, Olsen et al. [5] investigated the Danish version of ANL, demonstrating its correlation with hearing loss (Pure Tone Audiometry—PTA—in the best ear). Despite this, the correlation was weak, it only occurred in the first ANL session, and the ANL procedure was not standardized; in fact, the test was conducted with the aid of headphones, analyzing each ear independently, and it was striking that the ANL masker babble spectrum did not match the spectrum of the Danish ANL running speech.

We therefore conducted a multicenter study to evaluate the impact of the masker babble spectrum on the ANL test with a specific focus on the relationship between hearing loss and ANL results. The aim of the study is to define if the variations observed in ANL test results has a correlation with the type of masking babble speech noise used during the test, which was performed in three different languages in order to define if this masking effect could be modified by the different speech spectra of the different languages.

## 2. Materials and Methods

### 2.1. ANL Speech Material

Francart, Wouters et al. (KU–Leuven University—Belgium) developed 8 ANL files for the Amplifon Centre for Research and Studies, in which standard content was used as running speech and translated into 8 languages, read by a professional native speaker. The recording was performed at 25 dB FS (dB full scale) to ensure a good dynamic range and sound quality, and the average energy was kept at a constant speed. As ANL masker babble, “Auditec multitalker matched babble 20 talkers” was used. This babble file was speech-weighted to match the long-term spectrum of the specific ANL language version [6].

**Figure 1:**
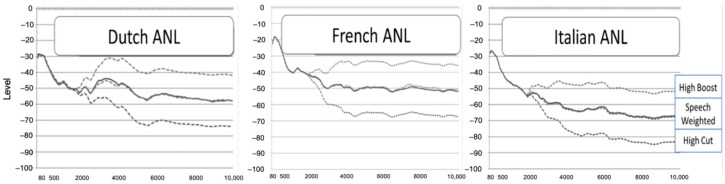
The long-term spectrum of the 3 language versions of the ANL test. High Boost masker babble files: top dotted lines; High Cut masker babble files: bottom dotted lines; speech-weighted masker babble files: middle dotted lines; running speech files: full lines. dBFS: dB Full Scale.

For this study, we created two extra masker babble signals for each language version, one where the spectrum was reduced from 2 kHz onwards (High Cut) and one version where the spectrum was increased from 2 kHz onwards (High Boost). This was performed for the Dutch (S1), French (S2), and Italian (S3) ANL versions; see Figure 1.

### 2.2. Equipment and Procedures

Audiometric thresholds, age, gender, and ANLs with 3 different masker babble files were collected for each listener in each of the three medical centers involved in the study.

ANL was performed in Free-Field, using one loudspeaker at 0° and at a one-meter distance from the listeners. The audiometer, Otometrics Aurical Aud, was calibrated according to ISO 8253-3:2012 [7], Acoustics—Audiometric test methods—Part 3: Speech audiometry, and calibration was checked at the listener’s position before the experiment.

Participants were given the same instructions across the three centers. Instructions for the MCL level were written as follows: “I will play a story for you. I want you to signal me to increase/decrease the story to determine your most comfortable listening level. I will instruct you along the way. Ask me to increase or decrease the level of the story to a comfortable listening level for you. Imagine I am the volume control of your television set, and you want the volume to be adjusted so you can hear the television at a comfortable level all evening”.

The instructions for assessing the BNL level were: “You will listen to the same story with background noise of several people talking at the same time. Tell us to adjust the noise to the MAXIMUM level that you would be willing to “put up with” for a long time while following the story”.

Pure-tone audiometry was tested using an Optometric Aurical Aud with TDH 39 headphones with Silent Caps. The audiometers were calibrated according to ISO 8253-1:2010 standard [7],: Pure-tone air and bone conduction audiometry.

### 2.3. Study Population

This study involved three medical centers: Thomas More University College, Antwerp, Belgium (Dutch), Marie Haps University College, Brussels, Belgium (French), and the University of Milan, Milan, Italy (Italian). A total of 198 patients were involved in the study, subdivided as follows:

Dutch: 73 native Dutch (S1)-speaking subjects, divided into 3 subgroups:

- Group A: 25 hearing-impaired, aged subjects, reporting a “hearing problem”, with a Fletcher index higher than 20 dBHL. The average age was 61.4 ± 18.08 s.d. years, with 40% being female and the average Fletcher index of the best ear being 35 ± 11.5 s.d. dBHL;
- Group B: 24 control subjects, with the same age and gender as group A, reporting “no hearing problems”. The average age was 62.1 ± 16.52 s.d. years, with 40% being female and the average Fletcher index of the best ear being 11 ± 8.82 s.d. dBHL;
- Group C: 24 young normal hearing subjects, with a Fletcher index lower than 20 dBHL for each ear; the average age was 22.8 ± 10.7 s.d. years, with 80% being female and the average Fletcher index of the best ear being 6 ± 9.1 s.d. dBHL.

French: 90 native French (S2)-speaking subjects, divided into 3 subgroups:

- Group A: 30 hearing-impaired aged subjects, reporting hearing problems, with a Fletcher index higher than 20 dBHL. The average age was 70.9 ± 14.81 s.d. years, with 53% being female and the average Fletcher index of the best ear equal being 33 ± 12.19 s.d. dBHL;
- Group B: 30 control subjects, stating that they had no hearing problems. The average age was 57.5 ± 21.6 s.d. years, with 43% being female and the average Fletcher index of the best ear equal to 16 ± 3.89 s.d. dBHL;
- Group C: 30 young normal hearing subjects, with a Fletcher index that was lower than 20 dBHL for each ear; the average age was 24.8 ± 14.7 s.d. years, with 47% being female and the average Fletcher index of the best ear being 7 ± 12.3 s.d. dBHL.

Italian: 36 native Italian (S3)-speaking, hearing-impaired, aged subjects. They all reported having a hearing problem, and they all had a Fletcher index higher than 20 dBHL; the average age was 73.5 ± 14.9 s.d. years, with 50% being female and the average Fletcher index of the best ear equal to 50 dBHL (Group A). Group B and C were not included because they had been tested previously in another setting which is not comparable to providing the same results as Group A.

### 2.4. Statistical Procedures

For the statistical analysis, we used Graph Pad Prism 6 for Windows. The median ANL values were utilized to perform a comparison among the three centers, given that the data distribution was uneven at multiple sites. In order to take into account the unevenness of the ANL test results given the different masker babble spectra used, we used the paired t-test for data with a normal distribution and the Wilcoxon matched-pairs signed rank test for data that did not have a normal distribution. To evaluate the normality of the distribution, we used the D’Agostino and Pearson omnibus normality test. The correlation was calculated with the Pearson correlation test for data with a normal distribution and with the non-parametric Spearman correlation test for data that did not have a normal distribution.

This study received approval from the Ethical Committee of Fondazione IRCCS Ca’ Granda Ospedale Maggiore Policlinico of Milan, Italy. Informed consent was obtained from all subjects involved in the study. (Protocol N. 478-2016bis). Patients’ anonymity has been guaranteed.

## 3. Results

### 3.1. ANL for the Different Masker Babble Types

**Figure 2:**
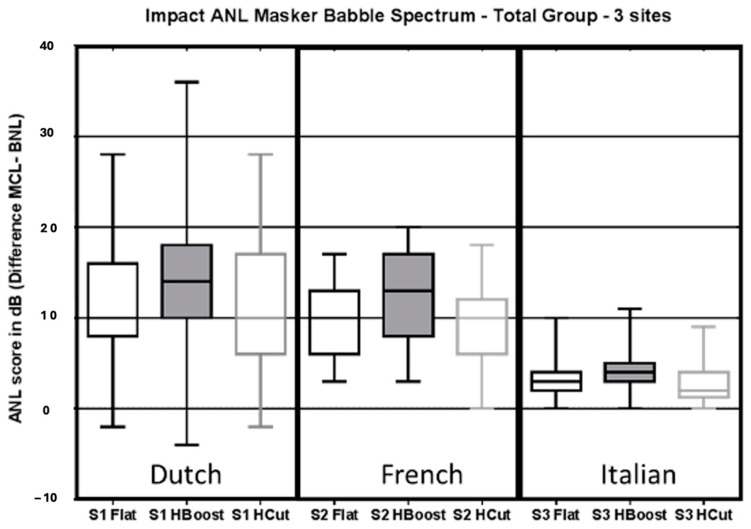
Box and whisker plot (representing the median value, the 25th and 75th percentiles, and the max and minimum values) with the ANL results for the total population at the 3 sites with different ANL masker types (Matched noise: black line with white filling; High Boost noise: black line with grey filling; High Cut noise: grey line with white filling).

As reported in Figure 2, considering the total population for the 3 centers, ANL with High Boost noise was significantly higher (worse) than ANL with Matched noise median ANL values: 14 (Dutch), 12 (French), and 4 (Italian); (*p*-value S1: <0.0001, S2: <0.0001, and S3: 0.0003). The overall median ANL values and the Inter-Quartile Range (IQR) of each sub-group are reported in Table 1.

ANL with High Boost noise was also significantly higher (worse) than ANL with High Cut noise in all languages (*p*-values < 0.05). ANL with High Cut noise provided similar results to the Matched noise in Dutch and French (*p*-values > 0.05), whereas in the Italian language, ANL with High Cut noise provided significantly lower (better) results than ANL with Matched noise (*p*-value 0.002).

Considering the subgroups, we found that in Dutch and French:

In Group A, ANL with High Boost noise was significantly higher than ANL with Matched noise (*p*-values < 0.05) and was significantly higher than ANL with High Cut noise (*p*-values < 0.05); in contrast, there was no difference between ANL with High Cut noise and ANL with Matched noise (*p*-value > 0.05).

In Group B, ANL with High Boost noise was also significantly higher than ANL with Matched noise (*p*-values < 0.05) and significantly higher than ANL with High Cut noise in French (*p*-value < 0.0001), while there was no difference between ANL with High Cut noise and ANL with Matched noise (*p*-values > 0.05).

In Group C, ANL with High Boost noise was significantly higher than ANL with Matched noise (*p*-values < 0.0001) and was significantly higher than ANL with High Cut noise (*p*-values < 0.0001). Furthermore, ANL with High Cut noise was significantly lower (better) than ANL with Matched noise (*p*-values < 0.05). See Table 2 for the detailed *p*-values.

### 3.2. ANL and Age

For the total population, none of the ANL values correlated with age at any of the centers, as shown in detail in Table 3.

### 3.3. ANL and Gender

For the total population, a weak correlation (Spearman *p* = 0.047/r = 0.24) was found in Dutch (Antwerp—Site 1) for ANL with High Boost and gender. The other ANLs did not correlate with gender. At the other two sites, no correlation was found (Table 3).

### 3.4. ANL and Hearing Loss

In Dutch, no correlation between ANL and hearing loss was found. In French, a weak correlation was found between ANL with High Cut noise and the Fletcher index of the worst ear (Spearman *p* = 0.034/r = 0.22). In Italian, a weak correlation was found between ANL with Matched noise and the Fletcher index of the best ear (Pearson *p* = 0.038/r = 0.35) and between ANL with High Boost noise and the Fletcher index of the best ear (Pearson *p* = 0.029/r = 0.36). See Table 2 for details.

## 4. Discussion

The primary application of the ANL test is to objectively quantify noise tolerance in hearing-impaired patients. This feature makes it a valuable parameter in the evaluation of patients prior to hearing aid use [2,8]. Evaluation of the background noise level in these patients is important when setting the parameters in hearing aids, such as noise reduction circuit, microphone sensitivity, and gain [9,10]. This is especially true for those patients who are unwilling to accept noise, helping to reduce the rejection rate of hearing aids; therefore, it can predict the impact of digital noise reduction (DNR) when using hearing aids [3,4,11].

For as much as it is known in the literature at present, ANL results do not seem to be influenced by listener characteristics such as gender, pure-tone average, or language spoken [12,13]. The results of our study are consistent with these data: ANL values did not show any correlation with gender, hearing acuity, and language. Recently, Shetty et al. observed that listening effort increases with age, especially in noisy environments. Despite this, our data did not show any correlation between ANL and age [14].

In 2006, Freyaldenhoven et al. investigated how the type of background noise affects ANL outcomes in normal hearing patients [15]. In accordance with their findings, we observed that the type of masking noise influenced the ANL outcome in all three different languages analyzed. In particular, High Boost noise showed higher values of ANL, regardless of any other patient characteristic considered. The worsening of High Boost noise could be attributed to the difficulties of consonant detection in noise in all of the languages. Likewise, High Cut noise provided significantly better values compared to the Matched noise in the Italian language. Since the Italian speaking frequency is much more targeted on the 2–4 kHz frequencies, we could speculate that this difference is attributable to the different speaking frequency ranges of these three languages. With High Cut noise, in French and Dutch, only young, normal hearing subjects (Group C), who are those more able to detect high frequencies, slightly performed better than with Matched noise, thus supporting this hypothesis.

To the authors’ knowledge, this is the first study analyzing the different masking noise effects on ANL values in normal hearing and impaired subjects. The results highlight the ever-worsening contribution of high frequencies in masking noise in all subjects.

Conversely, in 2007, Pyler et al. varied the speech and noise stimuli in ANL with a low-pass filter at 2.0 kHz and 6.0 kHz, showing that ANL was significantly poorer when the speech and noise stimuli were low-pass filtered at 2.0 kHz in relation to the 6.0 kHz condition [16]. Furthermore, the results show that ANL values were not significantly affected by the hearing sensitivity of the listeners. These data are in contrast with what was previously reported in the literature, which showed the sensitivity of ANL in diagnosing hearing loss [5]. Recruitment differences could be a possible explanation for this finding.

The preliminary results of this study may allow us to think that the introduction of high frequencies in noise is the most important condition affecting ANL results, regardless of the grade of hearing impairment. The burden brought by high frequencies in making noise less tolerable by patients, and thus in compromising the ANL value, should be taken into account considering that environmental noise can widely differ in frequency range. This could be a point for further investigation when talking about the noise reduction circuits of hearing aids.

## 5. Conclusions

Acceptable Noise Level is an objective test to quantify noise tolerance in both hearing-impaired patients and healthy patients. The results do not vary in relation to patient characteristics such as age or gender. As long as standardized protocols and speech-weighted babble files are used, the language spoken does not influence ANL values; thus, its application can provide objective results in predicting hearing aid use worldwide. Varying the spectrum of the masker babble on the ANL significantly influences the test values when high frequencies are introduced to the noise stimuli. Further investigations should focus on the weight brought by high frequencies in making noise less tolerable for patients who are willing to use hearing aids.

## Figures and Tables

**Table 1:** The median values and Inter-Quartile Range of the ANL in dB for the different sites, the different ANL masker types, and the different subgroups: A: hearing-impaired subjects; B: controls; C: normal hearing subjects.

		Median ANL (dB) (IQR)		
Group	Masker Babble Spectrum	Dutch	French	Italian
	Matched	10 (8–16)	9 (5–13)	3 (2–4)
Total	High Boost	14 (10–18)	12 (8–16)	4 (3–5)
	High Cut	10 (6–17)	8.5 (6–12)	2 (1.25–4)
	Matched	10 (8–16)	10 (3–10)	
A	High Boost	16 (8–18)	12.5 (3–12.5)	
	High Cut	12 (7–18)	10 (6.75–13)	
	Matched	11 (8–16)	8.5 (5–13)	
B	High Boost	13 (8.5–16)	9.5 (7–17)	
	High Cut	11 (6–21.5)	8 (4.75–12)	
	Matched	10 (8–18)	9.5 (4–13.25)	
C	High Boost	16 (10.5–23.5)	12 (7.75–15.25)	
	High Cut	8 (6–14)	7.5 (4.75–12.25)	

**Table 2:** Statistical comparison of the different ANL results based on the masker type for the different sites and for the different subgroups: A: hearing-impaired subjects; B: controls; C: normal hearing subjects. HB: High Boost; HC: High Cut; ns: not statistically significant (*p*-value > 0.05).

		*p*-Value		
Group	Masker Babble Spectrum	Dutch	French	Italian
	HB vs. Matched	0.0001	0.0001	0.0003
Total	HB vs. HC	0.0002	<0.0001	<0.0001
	HC vs. Matched	Ns	ns	0.02
	HB vs. Matched	0.03	<0.0001	
A	HB vs. HC	0.008	<0.0001	
	HC vs. Matched	Ns	ns	
	HB vs. Matched	0.06	<0.0001	
B	HB vs. HC	Ns	<0.0001	
	HC vs. Matched	Ns	ns	
	HB vs. Matched	<0.0001	<0.0001	
C	HB vs. HC	<0.0001	<0.0001	
	HC vs. Matched	0.008	0.02	

**Table 3:** Correlation *p*-values for the 3 sites between the 3 types of ANL and age, gender, and hearing loss (Fletcher index and Fletcher high index) for the best (B) and the worst (W) ear. Black font: Pearson *p*; grey font: Spearman *p*. Note that the *p*-values * that are underlined and in bold font represent significant *p*-values.

		Correlation (*p*-Value)		
	Masker Bubble Spectrum	Dutch	French	Italian
	Matched	0.92	0.64	0.09
Age	High Boost	0.15	0.83	0.21
	High Cut	0.26	0.43	0.32
	Matched	0.64	0.64	0.27
Gender	High Boost	**0.047** *	0.83	0.19
	High Cut	0.97	0.43	0.31
	Matched	0.69	0.81	**0.038** *
Fletcher Index B	High Boost	0.38	0.85	**0.029** *
	High Cut	0.72	0.16	0.08
	Matched	0.67	0.89	0.27
Fletcher High B	High Boost	0.12	0.88	0.41
	High Cut	0.63	0.28	0.72
	Matched	0.77	0.32	0.22
Fletcher Index W	High Boost	0.47	0.42	0.21
	High Cut	0.75	**0.034** *	0.44
	Matched	0.85	0.24	0.46
Fletcher High W	High Boost	0.24	0.56	0.66
	High Cut	0.56	0.05	0.97

## Data Availability

The data presented in this study are available from the corresponding author upon request due to our privacy policy.
